# A stepped wedge cluster randomized control trial to evaluate the implementation and effectiveness of optimized initiatives in improving quality of care for ST segment elevation myocardial infarction in response to the COVID-19 outbreak

**DOI:** 10.1186/s13012-021-01107-1

**Published:** 2021-04-12

**Authors:** Shuduo Zhou, Xuejie Dong, Fangjing Liu, Yan Zhang, Dahai Yue, Qiang Zhou, Yinzi Jin, Zhi-Jie Zheng

**Affiliations:** 1 Department of Global Health, School of Public Health, Peking University, 38 Xue Yuan Road, Haidian District, Beijing, 100191 China; 2 Institute for Global Health, Peking University, Beijing, China; 3 Institute of Cardiovascular Disease, Peking University First Hospital, Beijing, China; 4 University of Maryland, College Park, USA; 5 Shenzhen Center for Prehospital Care, Shenzhen, China

**Keywords:** ST segment elevation myocardial infarction, Quality-improved, RE-AIM, CFIR, Optimized, COVID-19, China

## Abstract

**Background:**

The National Chest Pain Center Accreditation Program (CHANGE) is the first hospital-based, multifaceted, nationwide quality improvement (QI) initiative, to monitor and improve the quality of the ST segment elevation myocardial infarction (STEMI) care in China. The QI initiatives, as implementation strategies, include a bundle of evidence-based interventions adapted for implementation in China. During the pandemic of coronavirus disease 2019 (COVID-19), fear of infection with severe acute respiratory syndrome coronavirus 2, national lockdowns, and altered health care priorities have highlighted the program’s importance in improving STEMI care quality. This study aims to minimize the adverse impact of the COVID-19 pandemic on the quality of STEMI care, by developing interventions that optimize the QI initiatives, implementing and evaluating the optimized QI initiatives, and developing scale-up activities of the optimized QI initiatives in response to COVID-19 and other public health emergencies.

**Methods:**

A stepped wedge cluster randomized control trial will be conducted in three selected cities of China: Wuhan, Suzhou, and Shenzhen. Two districts have been randomly selected in each city, yielding a total of 24 registered hospitals. This study will conduct a rollout in these hospitals every 3 months. The 24 hospitals will be randomly assigned to four clusters, and each cluster will commence the intervention (optimized QI initiatives) at one of the four steps. We will conduct hospital-based assessments, questionnaire surveys among health care providers, community-based household surveys, and key informant interviews during the trial. All outcome measures will be organized using the RE-AIM (reach, effectiveness, adoption, implementation, maintenance) framework, including implementation outcomes, service outcomes (e.g., treatment time), and patient outcomes (e.g., in-hospital mortality and 1-year complication). The Consolidated Framework for Implementation Research framework will be used to identify factors that influence implementation of the optimized QI interventions.

**Discussion:**

The study findings could be translated into a systematic solution to implementing QI initiatives in response to COVID-19 and future potential major public health emergencies. Such actionable knowledge is critical for implementors of scale-up activities in low- and middle-income settings.

**Trial registration:**

ChiCTR 2100043319. Registered on 10 February 2021

**Supplementary Information:**

The online version contains supplementary material available at 10.1186/s13012-021-01107-1.

Contributions to the literature

- This study will be the first to identify multilevel factors that emerge within the context of COVID-19 and influence implementation of the interventions to improve the quality of care for acute cardiac events. The findings will provide implications for where adjustments to the interventions could be made in response to COVID-19 and other potential pandemics or health emergencies.
- This study will provide a novel model to measure complex interventions in real-world health care system settings, including measures of clinical effectiveness and implementation strategies for quality improvement initiatives of acute cardiac care, by using a stepped wedge cluster randomized control design, under a modified RE-AIM (reach, effectiveness, adoption, implementation, maintenance) framework.
- The outcomes of this study will generate actionable knowledge about process outcomes and feasibility, including core components that are transferrable, and where local adaptation is needed for scale-up activities in low- and middle-income settings, to improve quality of care for acute cardiac events in response to COVID-19 and other potential pandemics or health emergencies.

## Background

The coronavirus disease 2019 (COVID-19) pandemic requires enormous deployment of health care resources worldwide. Fear of infection with severe acute respiratory syndrome coronavirus 2 (SARS-CoV-2), national lockdowns, and altered health care priorities can compromise the quality of care for non-COVID-19 diseases. The downstream effects have been observed in the acute care sector, which has been oriented toward a “war footing” for COVID-19 through major reorganization of emergency care facilities. This is particularly true for China and other low- and middle-income countries, where financial, technical, and staff resources are limited.

Importance of improving quality of care for acute cardiac events in China

ST segment elevation myocardial infarction (STEMI) is the deadliest and most time-sensitive acute cardiac event. Poor quality of care for patients with STEMI contributes to increased mortality from cardiovascular diseases, which is the leading cause of death in China. STEMI cases require rapid coordination of care beginning at the time of symptom onset. Percutaneous coronary intervention (PCI) within 120 minutes from onset is the typically recommended treatment, according to American College of Cardiology/American Heart Association (ACC/AHA) guidelines [1]. Despite the widespread promulgation and endorsement of these guidelines and the strong evidence base underpinning many guideline recommendations, their translation into clinical practice remains suboptimal [2, 3]. On the basis of our prior study, the time from onset to PCI is approximately 291 minutes in China, and only 7% of patients receive timely PCI therapy. Therefore, reducing the treatment delay by organizing delivery of care on a regional basis is a priority for improving quality of STEMI care in China.

The Chinese Cardiovascular Association launched the National Chest Pain Center Accreditation Program (CHANGE) in January 2016. This is the first nationwide, hospital-based, multifaceted, continuous quality improvement (QI) initiative, with aims to monitor and improve the quality of care for acute cardiac events (ClinicalTrials.gov, NCT04014972). The QI initiatives, as implementation strategies, include a bundle of evidence-based interventions adapted for implementation in China. These are in line with global best practices: (i) accreditation of hospital-based chest pain centers (CPCs), (ii) establishing a unified data registry for quality monitoring and assessment, and (iii) providing ongoing quality review and feedback. The operational structure was developed at the start of the program (Appendix Figure 1). The program is made available to all secondary and tertiary hospitals, and hospitals continue to join the program in a staggered manner. With the support of the National Health Commission (NHC), based on the CPC Accreditation Criteria issued by the Chinese Society of Cardiology, the CHANGE program has implemented the accreditation and development of CPCs (Appendix Figure 2). The CHANGE program developed the China CPC Data Reporting Platform, a web-based, unified national database serving as a national surveillance system for monitoring, evaluation, and feedback regarding quality of care for reported STEMI events (Appendix Table 1). As of June 30, 2019, there were 2,885 hospitals across 31 provinces of China and a total of 463,827 patients with a STEMI diagnosis enrolled in the program. Our preliminary analysis using baseline and quarterly data during January 2016 and June 2019 demonstrated that the QI initiatives can shorten onset to reperfusion times and subsequently reduce in-hospital mortality.

Challenges to quality improvement initiatives in response to COVID-19

The COVID-19 pandemic is challenging population health and health care systems in unprecedented ways, and the effects will last for decades to come [4]. Most countries have implemented stringent infection-control measures, including but not limited to social distancing measures, emergency infection protocols instituted in hospitals to contain COVID-19, and adjustment of clinical services. The response to COVID-19 can compromise rapid triage and may impact optimal treatment delivery for patients with acute cardiac events. Several studies have reported substantial declines in the number of patients presenting with acute cardiac events as well as the number of emergency coronary procedures, in both high-income and low- and middle-income countries [5–7]. Preliminary available data from the CHANGE program show that hospital admissions among patients with STEMI decreased by more than 50% in February 2020 compared with the same period in 2019. The time from onset to PCI increased from 237 minutes in February 2019 to 342 minutes in February 2020.

There are two major public health concerns with respect to the care in STEMI and other acute cardiac events: (i) delays in presentation, and (ii) delays in treatment [8–13]. First, causes for late presentation for STEMI are likely multifactorial and may include patient fear of contracting an infection from within the health care system. The lack of knowledge compounded by fear of the SARS-CoV-2 make patients with STEMI much less likely to seek help. The emphasis on social distancing might have inappropriately convinced patients to avoid in-person health care. Proper education including STEMI awareness and COVID-19 knowledge is warranted during a pandemic [8, 9, 14]. In the CHANGE program, accredited CPCs in hospitals should follow the criteria for establishing a medical consortium of STEMI care. Hospitals should be partnered with community health centers to form an information-sharing and resource-management model. Health workers at community health centers conduct health management of STEMI patients and health education among community residents, which are part of the essential public health services in China. Thus, community-based education supervised by hospitals within a medical consortium should be established within routine services delivery, to inform the public that the CPCs remain fully operational and have stringent infection-control protocols in place during the pandemic.

Second, delays in treatment may be prolonged because the emergency infection protocols (e.g., COVID-19 screening after hospital arrival) could result in a considerable delay in timely treatment. Additionally, care providers may be subject to travel restrictions or be dispatched to the front lines in fight against COVID-19, leading to a shortage of health workers and systemic overload. For example, over 40,000 medical professionals from other parts of China have been sent to Wuhan, Hubei Province, the epicenter of the outbreak, thereby draining the available resources for other disease conditions. Stresses on health systems are also likely to affect critical supply chains for essential medicines and equipment. Thus, it is imperative to improve the health care system’s ability to maintain care coordination for operational integrity with respect to patients with STEMI. A balance must be struck between identifying appropriate patients for PCI intervention for STEMI, regardless of their COVID-19 status, and maintaining the safety of health care workers who may be exposed to the virus as well as minimizing contamination of cardiac catheterization laboratory facilities [15, 16]. Prior studies have shown that a dedicated regional coordinator in charge of implementing systematic improvements within hospitals and EMS agencies in the region could play a critical role in maintaining coordination of care [17].

It is therefore necessary that health care systems develop plans for optimizing the existing QI initiatives, to mitigate the negative impact of the COVID-19 outbreak and to protect patients with acute cardiac events from nosocomial infection and limited access to care. This is not simply a problem limited to a single specialty or condition but rather a problem that holds lessons in acute illness care.

Contributions to current theory practice and policy

Following the positive preliminary phase and considering the needs in response to COVID-19, the QI initiatives should be optimized, as recommended by the NHC [18]. In collaboration with NHC partners and CHANGE program staff, this study will design an updated implementation strategy for this optimization, to enhance the adoption, implementation, and sustainability of QI initiatives in the context of COVID-19. However, little is known regarding identification of the best policy options and solutions based on China’s real-world situations, to improve the quality of care in response to COVID-19. Furthermore, to our knowledge, no studies have focused on implementation strategies to provide generalizable data that can inform replication and scale-up activities [17, 19]. Therefore, this study will add evidence-based interventions to current QI initiatives, to develop optimized QI initiatives from a multidisciplinary research perspective. The main contributions of the proposed project include the following.

First, a Consolidated Framework for Implementation Research (CFIR) framework will be used to identify multilevel factors that emerge within the context of COVID-19 and influence implementation of the current QI interventions [20], and where adjustments and refinements to the intervention could be made in response to COVID-19.

Second, a novel model will be used to measure the implementation strategy of the optimized QI initiatives, using a stepped wedge cluster randomized control design, under a modified RE-AIM (reach, effectiveness, adoption, implementation, maintenance) framework [21]. The RE-AIM model is a practical tool that assesses complex interventions in real-world health care system settings, including measures of clinical effectiveness and implementation strategies. The stepped wedge design will provide more generalizable estimates than traditional study designs because it allows for regular data analysis and iterative dissemination of results—internally, to support programmatic improvements and with partners for embedded research practices.

Third, the outcomes of this study could be translated into a systematic solution for implementing the QI initiatives in response to COVID-19 and other potential pandemics or health emergencies. The synthesis of the CFIR framework and RE-AIM evaluation framework will enhance the traditional stepped wedge design by including measures of implementation fidelity, and will generate knowledge about process outcomes and feasibility including barriers and facilitators, core components that are transferrable, and where local adaptation is needed for replication in other settings [22]. This actionable knowledge is a critical need for implementors of scale-up activities in low- and middle-income settings.

## Methods

### Study aims

The overall goal is to minimize the untoward impact of the evolving COVID-19 outbreak on the quality of STEMI care by optimizing the implementation of QI initiatives. The specific objectives of the study include the following:

1. To develop hospital-based interventions that optimize the QI initiatives, adapted to China’s health care system.
2. To implement the optimized QI initiatives, and evaluate the clinical effectiveness and implementation strategy using measures of reach, adoption, implementation, and maintenance.
3. To develop strategic options in scale-up activities for the optimized QI initiatives under two scenarios, including the outbreak stage of a major public health emergency and the pandemic period.

### Study design

**Fig. 1:**
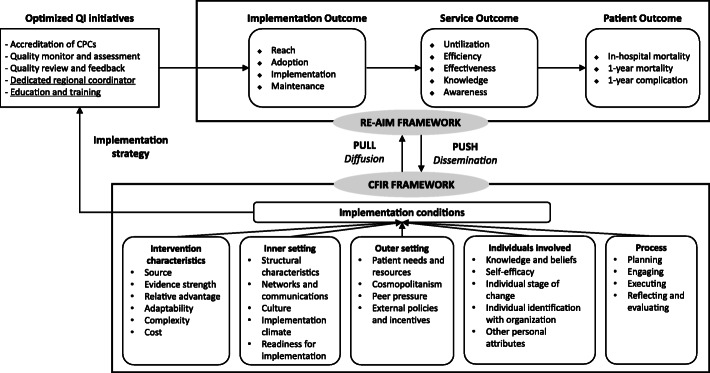
Theoretical model for the study

**Fig. 2:**
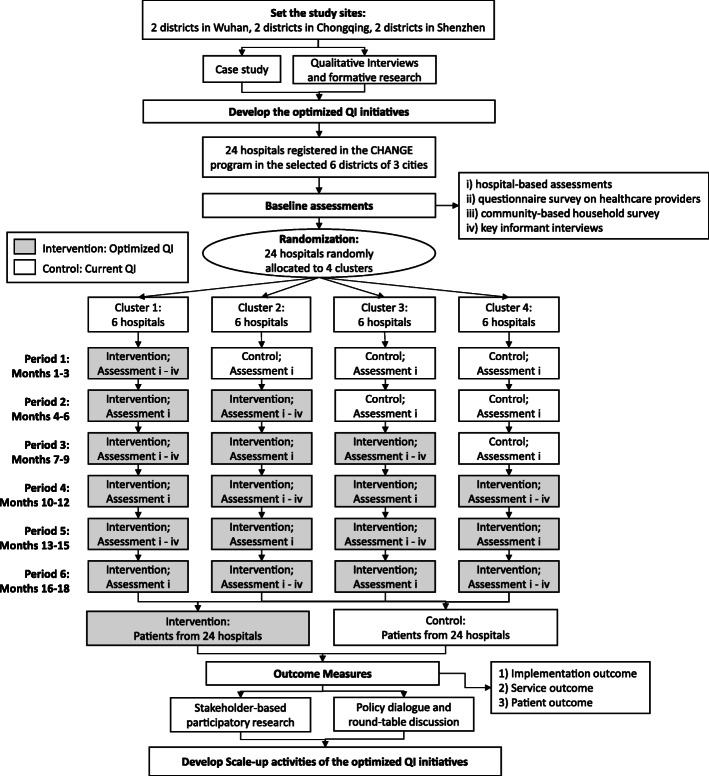
Study flow diagram

The theoretical model for this study is a synthesis of implementation and evaluation of the optimized QI initiatives under the theory of implementation science (Fig. 1). The RE-AIM framework will be used to evaluate the implementation strategy, in which three dimensions of outcomes will be included, namely implementation outcome, service outcome, and patient outcome. Effectiveness is measured according to service outcome and patient outcome. Reach, adoption, implementation, and maintenance are measured according to implementation outcomes. The CFIR framework will be used to identify the barriers and facilitators influencing the implementation strategy. Based on the theoretical model, we will use a stepped wedge cluster randomized control design to evaluate the implementation and effectiveness of the optimized quality improvement initiatives (Fig. 2).

### Setting and facility selection

The study will be conducted in cities of Wuhan, Suzhou, and Shenzhen of China. Study sites are selected by the NHC partners and the CHANGE program staff based on region, population size, economic level, traffic conditions, and representativeness of the prehospital model. The prehospital model varies across cities. In general, there are now at least three prehospital models delivering emergency medical services (EMS). The first type operates independently (e.g., Wuhan), the second is under the authority of emergency centers (e.g., Suzhou), and the third is delivered by the emergency department of hospitals (e.g., Shenzhen). Two districts will be randomly selected in each of the three cities. All hospitals registered in the CHANGE program in the six selected districts will be included; finally, this study will include 24 registered hospitals.

### Intervention

The intervention will be applied at the hospital level. Intervention hospitals will carry out the optimized QI initiatives, and hospitals that have not been enrolled in the intervention will conduct the current QI initiatives (Table 1). In terms of the current QI initiatives, hospitals need to go through the accreditation process to develop an accredited CPC. Accredited CPCs should follow the criteria for improving the quality of care of patients with STEMI [23]. The continuous QI activities require hospitals to report real-time clinical data for monitoring the CPC performance. Improvement in adherence to the guideline recommendations is facilitated through monthly, quarterly, and annually hospital-specific performance feedback reports [24]. The hospital-specific data are compared against a variety of internal and external benchmarks, including the temporal trend in performance and comparison points to regional or national performance thresholds [25]. In terms of the two added interventions among the optimized QI initiatives, the dedicated regional coordinator will be charged with coordinated care between community, hospitals, and EMS agency [26]. The training outreach targeting healthcare professionals will be conducted by hospitals, and the education targeting community residents will be conducted by community health centers [27].

**Table 1:** Optimized and current QI initiatives in the CHANGE program

QI initiatives	Details	Classification
(1) Accreditation of hospital-based CPCs	▪ The comprehensive center criteria are applicable to the comprehensive CPCs at tertiary hospitals; while the basic center criteria targets the basic CPCs at secondary hospitals. ▪ Both editions of the criteria include 5 dimensions of qualification: conditions of facilities, diagnosis and treatment process, integration of prehospital and hospital care, training and education, and real-time data reporting. ▪ Registered hospitals need to go through 3 stages including self-assessment, accreditation, and re-accreditation every 3 years, to develop an accredited CPC. ▪ The accreditation process is jointly led by the China CPC Headquarters, Regional Accreditation Offices, and Provincial-level CPC Alliances.	Current and optimized
(2) Quality monitor and assessment	▪ Accredited hospitals should continuously report data for monitoring and feedback. ▪ The indicators for measuring the CPC performance in the quarterly and annually benchmarked reports are developed by the China CPC Headquarters, based on the ACC/AHA Performance Measures and clinical practice guidelines. ▪ There are two sets of performance measures respectively for comprehensive and basic CPCs. ▪ Ranking of a CPC is calculated based on the percentile of each indicator and a weighted composite score. The score of 100, 80, 60, 40, 20, and 0 are for ranking the top 10%, 10–30%, 30–50%, 50–70%, 70–90%, and 90–100% of the measure among the entire accredited CPCs.	Current and optimized
(3) Quality review and feedback	▪ Improvement in adherence to the guideline recommendations is facilitated through monthly and quarterly hospital-specific performance feedback reports. ▪ The hospital-specific data are compared against a variety of internal and external benchmarks, including the temporal trend in performance and comparison points to regional or national performance thresholds. ▪ A series of regular meeting, QI analysis meeting, and case study meeting are carried out at least once every quarter for sharing of ‘best practice’ clinical support tools.	Current and optimized
(4) Dedicated regional coordinator	▪ Dedicated regional coordinator charged with implementing systematic improvements within every hospital and EMS agency will be assigned by city-level CPC Alliances. ▪ Dedicated regional coordinators will work with the local health bureau to promote the optimized QI initiatives. ▪ The work of dedicated regional coordinators will include synthesizing emergency infection protocols to contain COVID-19 with QI initiatives, coordinating care between community, hospitals, and EMS agency, maintaining the safety of healthcare workers, minimizing contamination of laboratory facilities. ▪ The work of dedicated regional coordinators will be supervised by the city-level CPC Alliances and the China CPC Headquarters.	Optimized
(5) Education and training activities	▪ The training on emergency infection protocols instituted in hospitals to contain COVID-19 targeting at healthcare professionals will be conducted by hospitals ▪ Education on STEMI awareness and COVID-19 knowledge targeting community residents will be conducted by community health centers, which are trained and supervised by hospitals within the medical consortium.	Optimized

*QI* Quality improvement; *CHANGE* National Chest Pain Center Accreditation Program; *CPC* Chest pain center; *EMS* Emergency medical services; *ACC/AHA* American College of Cardiology/American Heart Association; *COVID-19* Coronavirus disease 2019; *STEMI* ST segment elevation myocardial infarction

Sampling

In this study, we will conduct a rollout to registered hospitals every 3 months, which will be based on the pilot experience, local contextual factors, budgetary and feasibility considerations. Patients with STEMI in registered hospitals will be consecutively enrolled in five 3-month steps. No intervention will be applied in the first step among all registered hospitals. Hospitals will be randomly allocated to one of four clusters, with six hospitals in each cluster. Each cluster will commence the intervention at one of the four remaining steps. All hospitals will be on the intervention in the final step. Characteristics of hospitals with STEMI cases enrolled during January and June 2020 are shown in Table 2, by district (Table 2).

**Table 2:** Characteristics of sample hospitals, by district

City	District	Population	Land area (km^2^)	Number of hospitals	STEMI patients	In-hospital mortality
Wuhan	Jianghan	496,289	28.3	3	459	1.53
	Qiaoko	528,604	40.1	4	319	3.13
Suzhou	Gusu	957,500	83.4	4	167	3.86
	Kunshan	1,665,900	931.5	6	157	1.27
Shenzhen	Futian	1,633,700	78.5	4	250	6.00
	Luohu	1,039,900	78.7	3	229	4.37

*STEMI* ST segment elevation myocardial infarction

### Randomization

Randomization will be done centrally among all 24 hospitals before initiating the intervention in the first-cluster hospitals. The allocation codes will be concealed by the statistician separately and will be provided to CHANGE program staff who are responsible for initiation of the intervention. The cluster order for implementation at each step will be determined randomly by an external technical advisor using a random number generator. Randomization will occur 6 months prior to rollout of the intervention in the next cluster. This will enable blinding to the random order of clusters for the NHC partners and CHANGE program staff involved in implementation while also allowing for an annual 6-month planning stage prior to starting the intervention.

### Sample size

This study is powered to detect a change according to cluster in admissions and in-hospital mortality per 100 STEMI cases. A sample size of 1581 participants will provide 80% power to detect an estimated 15% reduction or greater in mortality from the estimated baseline of 3.18 per 100 STEMI cases, with an alpha of 0.05, intra-cluster correlation of 0.005, and 20% non-response rate. The effect size is a conservative estimate based on past pilot experience (ClinicalTrials.gov, NCT04014972).

Data will be collected via the China CPC Data Reporting Platform (http://data.chinacpc.org/). Registered hospitals are instructed to enroll consecutive patients admitted to hospitals with acute cardiac events, and real-time reporting, as required. To be eligible, we will collect data from patients who meet the following criteria: (1) age 18 years or older; (2) a discharge diagnosis of STEMI based on ischemic symptoms, ECG changes, or positive cardiac markers; (3) admitted via all kinds of modes including directly by self, via EMS, transferred in, or in-hospital.

### Project activities

Taking advantage of ongoing monitoring and evaluation of the quality of STEMI care and continuous data collection in the CHANGE program, this study will achieve the goals and objectives through the following tasks and approaches.

1. Task 1. Design of optimized QI initiatives

Specific activities

1. Summarize China’s current experience in improving the quality of STEMI care in response to COVID-19 and focusing on the selected cities.
2. Identify priority intervention areas in the quality of the care response to COVID-19.
3. Identify key players and actors and their roles in optimizing QI initiatives in China.
4. Develop hospital-based interventions based on the current QI initiatives.

Approaches

1. Case studies

We will carry out case studies in the three selected cities, and we will summarize practices in implementation of the current QI initiatives, analyze the development of health care systems and infection-control measures, and assess the demographic profile of the population and utilization of emergency and medical services.

2) Qualitative interviews and formative research

This study will draw upon a series of interviews (face-to-face and by telephone and email) and focus group discussions conducted with key informants from governmental and nongovernmental agencies, organizations, and institutions. The interviews and discussions will be based on the initial outputs produced in the pilot study and case studies. A list of questions will then be developed and submitted to the study organization for consultation, review, comments, and further clarification.

(2) Task 2. Implementation and evaluation of optimized QI initiatives

Specific activities

1. Implement the optimized QI initiatives in the 24 selected hospitals in a staggered manner.
2. Compare longitudinal changes in the quality metrics of STEMI care and assess the clinical effectiveness of the optimized QI initiatives.
3. Analyze changes in the measures of reach, adoption, implementation, and maintenance, to evaluate the implementation strategy of the optimized QI initiatives.
4. Identify barriers and facilitators contributing to implementation of the optimized QI initiatives.

Approaches

1. Stepped wedge cluster randomized control trial

This pragmatic design leverages the staggered rollout of the optimized QI initiatives and will facilitate the assessment of effectiveness and implementation strategy. The trial will include four clusters in which the optimized QI initiatives are implemented sequentially every 3 months. A local project office in each district will be set up to manage the process of implementation. The optimized QI initiatives will be sequentially implemented by cluster [28, 29], and baseline assessment will be conducted within 3 months prior to implementing the optimized QI initiatives (Table 3).

2) Evaluation of the optimized QI initiatives

**Table 3:**
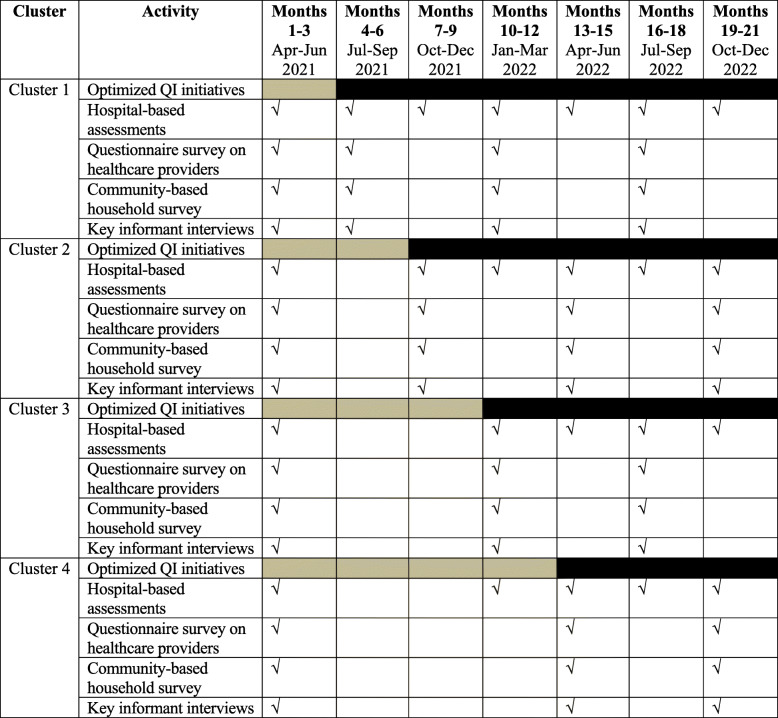
Optimized quality improvement (QI) initiatives, timeline, and data collection based on staggered implementation

The black portion in rows indicates the intervention phase and the gray portion indicates the control phase

To evaluate the effectiveness and implementation of the optimized QI initiatives, we will conduct a pragmatic hybrid type II effectiveness-implementation study, as this allows for simultaneous mixed-methods evaluation. All measures will be organized using a modified RE-AIM framework. Table 4 summarizes outcomes, data collection, and analysis plans organized by objective and adapted RE-AIM domains.

**Table 4:** Outcomes, data collection, and analysis plans organized by adapted RE-AIM domains

Outcomes	Indicators	Data source	Indicator definition
Service outcome	Number of admissions	Hospital-based assessments	The number of admissions for STEMI patients
	PCI rate	Hospital-based assessments	The proportion of STEMI patients who receive PCI
	Percentage of EMS transfer	Hospital-based assessments	The percentage of STEMI patients who are transferred by EMS agency
	Onset-to-FMC time	Hospital-based assessments	The time from onset to first medical contact of STEMI patients
	Door-to-balloon time	Hospital-based assessments	The time from arrival in hospital to PCI of STEMI patients
	FMC-to-device time	Hospital-based assessments	The time from first medical contact to PCI of STEMI patients
	Percentage of onset-to-FMC time ≤ 60 min	Hospital-based assessments	Percentage of STEMI patients with the time from onset to first medical contact ≤ 60 min
	Percentage of Call-to-EMS time ≤ 15 min	Hospital-based assessments	Percentage of STEMI patients with the time from calling EMS agency to ambulance arrival ≤ 15 min
	Percentage of Door-to-balloon time ≤ 60 min	Hospital-based assessments	Percentage of STEMI patients with the time from arrival in the hospital to PCI ≤ 60 min
	Percentage of FMC-to-device time ≤ 90 min	Hospital-based assessments	Percentage of STEMI patients with the time from first medical contact to PCI ≤90 min
Patient outcome	In-hospital mortality	Hospital-based assessments	Proportion of STEMI patients discharged death
	1-year mortality	Community-based household survey by telephone	Death rate of the STEMI patients within 1 year after hospitalization
	1-year complication rate	Community-based household survey by telephone	Incidence rate of new vascular events of STEMI patients within 1 year after hospitalization
Implementation outcome—reach	Number of patients visits	Community-based household survey	Proportion of the STEMI patients reporting care at a health facility
	Number of residents receiving health education	Community-based household survey	Number of individuals who receive education on STEMI awareness and COVID-19 knowledge
	Training the QI initiatives for Health providers	Questionnaire survey on healthcare providers	Number and proportion of the healthcare providers who receive the QI initiatives training
Implementation outcome —adoption	Community engagement	Community-based household survey	Number of community residents attending the optimized QI initiatives
	Health providers engagement	Questionnaire survey on healthcare providers	Number of health providers attending the optimized QI initiatives
	Behavior change of healthcare providers	Questionnaire survey on healthcare providers	Change score of healthcare providers in compliance with protocol of clinical guidelines
	Health literacy change of residents	Community-based household survey	Change score of health literacy related to STEMI awareness and COVID-19 knowledge of individuals
	Attitude of health facility directors	Key informant interviews	Degree of acceptance of the optimized QI initiatives by directors from hospitals and EMS agency
Implementation outcome—implementation	Fidelity	Key informant interviews	Degree that the optimized QI initiatives are implemented as planned in original protocol
	Feasibility	Key informant interviews	Extent that the optimized QI initiatives can be carried out in a specific setting
	Outer context	Key informant interviews	Macro-level external factors including social, funding, and leadership
	Inner context	Key informant interviews	Micro-level internal factors including NHC partnership, the programmatic staff, feedback, hospitals, EMS agency, community, household, and individual level
Implementation outcome —maintenance	Sustainable of the effectiveness	Key informant interviews	Views on maintaining effectiveness from policy makers, health facility directors, healthcare providers, and residents
	Satisfactory of stakeholders	Key informant interviews	Satisfactory on effectiveness and implementation strategy of the optimized QI initiatives of policy makers, health facility directors, healthcare providers, and residents
	Financial sustainable	Key informant interviews	Views on funding and return on investment from policy makers and health facility directors
	Institutionalization of interventions	Key informant interviews	Core components which are transferrable and where local adaptation is needed for replication in other settings

*RE-AIM* Reach, effectiveness, adoption, implementation, maintenance; *PCI* Percutaneous coronary intervention; *STEMI* ST segment elevation myocardial infarction; *EMS* Emergency medical services; *FMC* First medical contact; *QI* Quality improvement; *COVID-19* Coronavirus disease 2019; *NHC* National Health Commission

To measure outcomes, four components of the proposed work at each cluster will be conducted: i) hospital-based assessments, ii) questionnaire surveys of health care providers, iii) community-based household surveys, and iv) key informant interviews. Further details about each study component are described below, with Table 3 summarizing the timeline.

i. Hospital-based assessments

The assessments will be carried out at hospital level by collecting data via the China CPC Data Reporting Platform. Data elements for quality-of-care metrics will be selected based on the ACC/AHA clinical data standards. The assessments will be conducted in each cluster at baseline, at the start of the trial, and at subsequent 3-month intervals until the end of the trial.

ii. Questionnaire surveys of health care providers

Health care providers’ experiences and perceptions will be assessed via questionnaire surveys, to inform the consideration of reach and adoption efforts. Questionnaire survey data will be collected from dedicated regional coordinators, cardiologists, medical staff in emergency departments, and hospital managers. The assessments will be conducted in each cluster at baseline, at the start of the trial, and at subsequent 6-month intervals until the trial ends.

iii. Community-based household surveys

Household surveys will be conducted at the community level using a self-designed questionnaire to inform consideration regarding patient outcomes, as well as reach and adoption efforts. Surveys will target patients with STEMI and the general catchment population. The surveys will be conducted in each cluster at baseline, at the start of the trial, and at subsequent 6-month intervals until the trial ends.

iv. Key informant interviews

Qualitative interviews will be completed with key informants to assess adoption and maintenance. Key informants will include implementing partners in the program and local governments, as well as clinical and administrative employees in hospitals and EMS agencies. The key informant interviews will be conducted in each cluster at baseline, at the start of the trial, and at subsequent 6-month intervals until trial ends.

(3) Task 3. Development of scale-up activities in the optimized QI initiatives

Specific activities

1. Identify barriers and facilitators to program implementation fidelity and feasibility while also documenting contextual factors.
2. Investigate core requirements for implementing the optimized QI initiatives in response to COVID-19.
3. Identify gaps between the current experience in China’s practices of implementing the optimized QI initiatives.
4. Develop strategic and policy options for scale-up activities of the optimized QI initiatives under two scenarios, including the outbreak stage of a major public health emergency and the pandemic period.

Approaches

1. Stakeholder-based participatory research

Stakeholder-based participatory research will be conducted at the end of the stepped wedge cluster randomized control trial, to focus on partnerships, engagement, co-learning, and building on existing assets within China. Major stakeholder agencies and organizations will be invited to participate in this exercise. The exercise will help to identify factors influencing the implementation strategy using the CFIR framework, investigate core requirements for implementing the optimized QI initiatives in response to COVID-19, and identify gaps between the current experience in China’s practices in implementing the optimized QI initiatives according to the designed interview outline.

2) Policy dialogue and round-table discussion

We will translate the findings and results of this study into policy briefs and reports. Both electronic and paper-based briefs and internal policy analytic reports will be distributed through university think tank-based and extra-university channels to relevant decision-making bodies and agencies. A round-table discussion will be convened, to allow for feedback and comments on the results and findings of the study and provide opportunities for knowledge uptake among all stakeholders, decision makers, and executive entities, using push techniques to elicit pull by tailoring dissemination, to address the needs and concerns of decision makers.

### Statistical methods

1. Task 1. Design of the optimized QI initiatives

For Task 1, the analytic hierarchy process will be applied to identify the priority intervention areas in the quality-of-care response to COVID-19. A facilitated and structured interview matrix technique will be used to analyze the interview results for organizational analysis and strategic planning of priority settings. The structured interview matrix will follow a graded approach to collaboration involving discussion at three levels, using a three-step process: (1) interviews conducted by participants in the group; (2) small group deliberation; and (3) a facilitated, plenary discussion with the full group.

(2) Task 2. Implementation and evaluation of the optimized QI initiatives
(3) To investigate the effectiveness of the optimized QI initiatives with respect to quality metrics of STEMI care in response to COVID-19

Primary analysis will be performed according to the intention-to-treat principle. All analyses of outcomes will be at the individual level but will account for the clustering of patients at the hospital level. Comparisons of quality metrics between intervention and control participants will be conducted using the *t*-test and *χ*^2^ test. To analyze intervention effects, generalized estimating equation models will be used to account for the clustering within hospitals. The primary model will include a fixed effect for time and a binary variable for the effect of the intervention. The intervention effects will be summarized as the resulting odds ratios and difference of proportions for binary outcomes or mean differences for continuous outcomes. We will also conduct two-level generalized linear mixed models with patient and hospital as the first and second levels, respectively, using covariate-adjusted analyses. The model will also include the severity of the COVID-19 pandemic as a confounding factor. Appendix Table 2 depicts how the results will be displayed.

2) To examine the implementation strategy of the optimized QI initiatives using measures of reach, adoption, implementation, and maintenance in response to COVID-19

We will use a mixed-effects generalized linear model to compare pre-intervention to post-intervention proportions for each metric of reach and adoption, while adjusting for clustering at the hospital level and time and allowing for hospital-level estimates to be random effects; Appendix Table 3 shows how the results will be displayed. Evaluation of implementation and maintenance will be completed using questionnaires and interviews following the CFIR framework. The results of CFIR domains will complement quantitative data collected to evaluate implementation strategy and will assess emerging themes in identifying barriers and facilitators contributing to implementation of the optimized QI initiatives, to minimize adverse impacts of COVID-19 outbreak (Appendix Table 2).

(3) Task 3. Development of scale-up activities of the optimized QI initiatives

For task 3, the CFIR framework will be used to identify factors that may emerge in various contexts and that influence intervention implementation and effectiveness. We will use a template-analysis approach to code and organize our data for analysis by CFIR domain, including intervention characteristics, inner setting, outer setting, characteristics of individuals involved in implementation, and implementation process. Then, we will populate analytic matrices with the information for cross-case analysis of patterns in barriers and facilitators related to each of the program components. Our analytic matrices will facilitate simultaneous viewing of a large volume of data, to make between-practice comparisons and identify similarities, differences, and trends in how practices have experienced implementation.

## Discussion

The COVID-19 pandemic compromises the quality of care for the acute cardiac events. How to minimize the untoward impact of the evolving COVID-19 pandemic on the health care quality has been an imminent agenda for policymakers as well as researchers. This project will be the first to develop and implement hospital-based interventions that improve the quality of care for acute cardiac events in response to COVID-19, and to develop strategic options in scale-up activities for the quality improvement initiatives in response to COVID-19 and other potential pandemics or health emergencies. We have described our rationale, study design, and implementation strategy details regarding this stepped wedge cluster randomized control design.

### Feasibility of the project

With the support of the NHC, the project will be conducted in collaboration with the Peking University First Hospital and the Chinese Cardiovascular Association, which is committed to organizing and implementing the CHANGE program. Supervised by the Chinese Cardiovascular Association, the provincial- and city-level CPC Alliances manage and undertake implementation of the optimized QI initiatives at provincial and city levels, respectively. Data for hospital-based assessment will be collected via the China CPC Data Reporting Platform, which is managed by the Chinese Cardiovascular Association. Necessary personnel, including the project promotor, and dedicated regional coordinators in every hospital and EMS unit, will be assigned by the city-level CPC Alliances. The local project office in each district will be set up to manage personnel, information, and institutional coordination. The project promotor in each district will work with the local health bureau to promote implementation of the optimized QI initiatives, and they will be supervised by the city-level CPC Alliances.

Four specific activities will be conducted to mitigate potential risk during all procedures of this project, through effective technical and financial management and to coordinate collaborative functions. First, we will develop effective coordination mechanisms at all stages, to achieve the project objectives and maintain communication between project partners. Second, we will ensure the overall administrative, legal, and financial management of the project, and each participant will have their own responsibility with regard to these areas. Third, documentation and reporting of the project activities, workshop results, preparation of guides, and progress/review reports will be included, to resolve unforeseeable issues that may occur during the project. Fourth, we should ensure that ethical issues are appropriately taken into account and in a timely manner; and administrative permissions should be obtained that are needed to start and maintain the project activities.

Monitoring of the research process will be conducted according to the tasks and approaches. The activities of monitoring are shown in the flowchart (Appendix Table 4). Quality control procedures will be carried out, to help implement the activities through effective technical and financial management. The plan for quality control procedures according to approaches and the potential risks is shown below (Appendix Table 5).

### Pilot study

We have performed a retrospective cohort study drawing on unified registered-hospital report data from the CHANGE program. We investigated the effectiveness of the current QI initiatives in terms of quality metrics of STEMI care during January 2020 and June 2020 by comparing the data at baseline and in the final quarter. We then compared changes in the quality metrics of STEMI care between January and June 2019 and between January and June 2020 using difference-in-difference analysis.

The primary results of the polit study are as follows. First, baseline and quarterly data during January 2016 and June 2019 demonstrated that implementing the current QI initiatives significantly improved many process indicators. For example, the onset-to-device time decreased from 291 minutes to 233 minutes, and the percentage of cases with FMC-to-device time≤ 90 minutes increased from 43.0% to 54.1% between baseline and the final quarter. Second, during January and July 2020, admissions of patients with STEMI decreased by 42%, compared with the same period in 2019. Baseline and quarterly data showed that door-to-balloon and the FMC-to-device median (q1, q3) time increased from 17.5 (10.0, 46.0) and 52.0 (12.0, 86.0) minutes to 34.0 (15.0, 48.0) and 63.0 (15.0, 94.0) minutes, respectively (*p* = 0.001, *p* = 0.005). The onset-to-FMC time accounted for more than 70% of the total delayed time. Third, compared with January to June 2019, many process indicators deteriorated from January to June 2020. For example, the proportion of patients admitted via EMS was only 4.2% in 2020, much lower than that in 2019 (9.3%, *p* = 0.013), and rate of PCI practice declined from 71.3% to 60.1% (*p* = 0.002).

The additional expected primary results of the pilot study will include the following: First, the CFIR framework will be developed to support evaluation of implementation of the current QI initiatives within the context of COVID-19, and to produce actionable evaluation findings intended to improve implementation in a timely manner; Second, In-depth interviews with key informants will be conducted to identify barriers and facilitators to implementation of the current QI initiatives that may emerge in the context of COVID-19, using the CFIR framework to guide data collection, coding, analysis, and reporting of actionable findings for the design of the optimized QI initiatives.

### Strengths and limitations

This study will provide contributions to the literature as follows. First, this study will take lead to identify multilevel factors that emerge within the context of COVID-19 and influence implementation of the interventions to improve the quality of care for acute cardiac events, using the CFIR framework. The findings will provide implications for where adjustments to the interventions could be made in response to COVID-19 and other potential pandemics or health emergencies. Second, the study design will provide a novel model to measure complex interventions in real-world health care system settings, including measures of clinical effectiveness and implementation strategies for quality improvement initiatives of acute cardiac care in response to COVID-19, by using a stepped wedge cluster randomized control design under an implementation science framework, the RE-AIM model. Third, the outcomes of this study will generate actionable knowledge about process outcomes and feasibility, including core components that are transferrable, and where local adaptation is needed for scale-up activities in low- and middle-income settings, to improve the quality of care for acute cardiac events in response to COVID-19 and other potential pandemics or health emergencies.

However, this project has at least two limitations. First, in a real-world pragmatic stepped wedge trial, there will be concerns related to confounding, bias, and temporal trends that may limit the validity of the findings. However, we will use a cluster randomized control design, which is widely considered the best design for causal inference. The pragmatic design will be chosen because it allows iterative dissemination of results internally, to support programmatic improvements; it is anticipated that the project will be beneficial and receipt of the intervention is the strong preference of all registered hospitals. Nevertheless, we will use a modest effect size, cluster randomization, and an analysis plan to mitigate these limitations.

Second, the voluntary participation of hospitals limits our generalization to hospitals not participating in this registry, although hospital recruitment remains ongoing. Willingness of enrolled hospitals to participate in the program may indicate a greater focus on quality improvements and thus may limit reproducibility at other hospitals. We investigated the clinical regulation rules in the sample hospitals and hospitals that have not participated in the program; we found that differences in the clinical pathways and health resource mobilization were not significant. One explanation for this is that most hospitals in China are public and follow similar regulations issued by the NHC. Nevertheless, we will minimize the limitations at baseline through site selection and randomization.

## Supplementary Information

**Additional file 1: **Appendixes for the manuscript text. **Appendix Figure 1.**The CHANGE Operational Structure. **Appendix Figure 2.** The Chest Pain Center Accreditation Workflow in the CHANGE program. **Appendix Table 1.**The CHANGE registry data elements. **Appendix Table 2.**Effect of the optimized QI intervention on quality metrics. **Appendix Table 3.**Comparison of reach and adoption of the optimized QI initiatives between pre-and post-intervention. **Appendix Table 4.**Tasks, approaches, and activities of monitoring of the project. **Appendix Table 5.**Approaches, risk description, and quality control procedures of the project

## Data Availability

Please contact the corresponding author for more information

## Abbreviations

QI: Quality improvement
CHANGE: National chest pain center accreditation program
CPC: Chest pain center
EMS: Emergency medical services
ACC/AHA: American college of cardiology/American heart association
COVID-19: Coronavirus disease 2019
STEMI: ST segment elevation myocardial infarction
RE-AIM: Reach, effectiveness, adoption, implementation, maintenance
PCI: Percutaneous coronary intervention;
FMC: First medical contact
NHC: National health commission
ICC: Intra-cluster correlation coefficient
CI: Confidence interval
SD: Standard deviation
