# Supplementary material for: A stepped wedge cluster randomized control trial to evaluate the implementation and effectiveness of optimized initiatives in improving quality of care for ST segment elevation myocardial infarction in response to the COVID-19 outbreak
Source: Implement Sci. 2021 Apr 12;16:38. doi: 10.1186/s13012-021-01107-1 (PMC8040354; doi:10.1186/s13012-021-01107-1)
Supplement: Supplementary file 1 — Additional file 1: Appendixes for the manuscript text. Appendix Figure 1.The CHANGE Operational Structure. Appendix Figure 2. The Chest Pain Center Accreditation Workflow in the CHANGE program. Appendix Table 1.The CHANGE registry data elements. Appendix Table 2.Effect of the optimized QI intervention on quality metrics. Appendix Table 3.Comparison of reach and adoption of the optimized QI initiatives between pre-and post-intervention. Appendix Table 4.Tasks, approaches, and activities of monitoring of the project. Appendix Table 5.Approaches, risk description, and quality control procedures of the project [file 13012_2021_1107_MOESM1_ESM.doc]

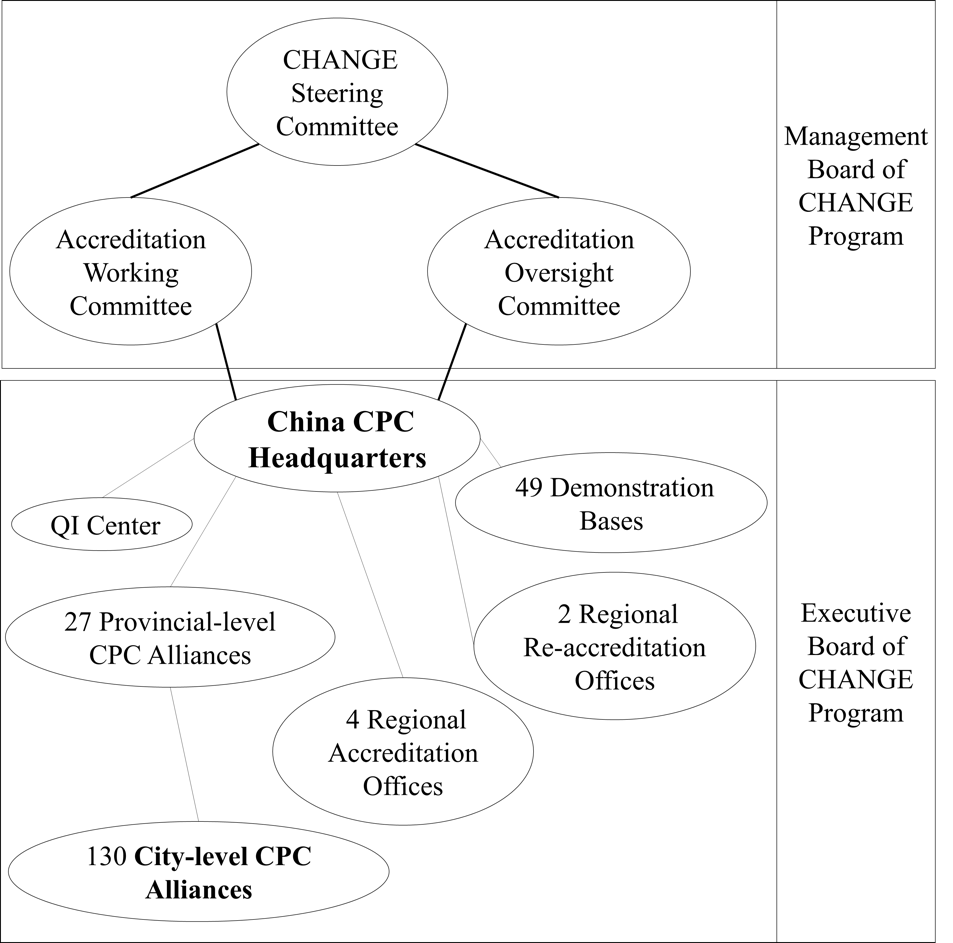


**Appendix Figure 1.** The CHANGE Operational Structure


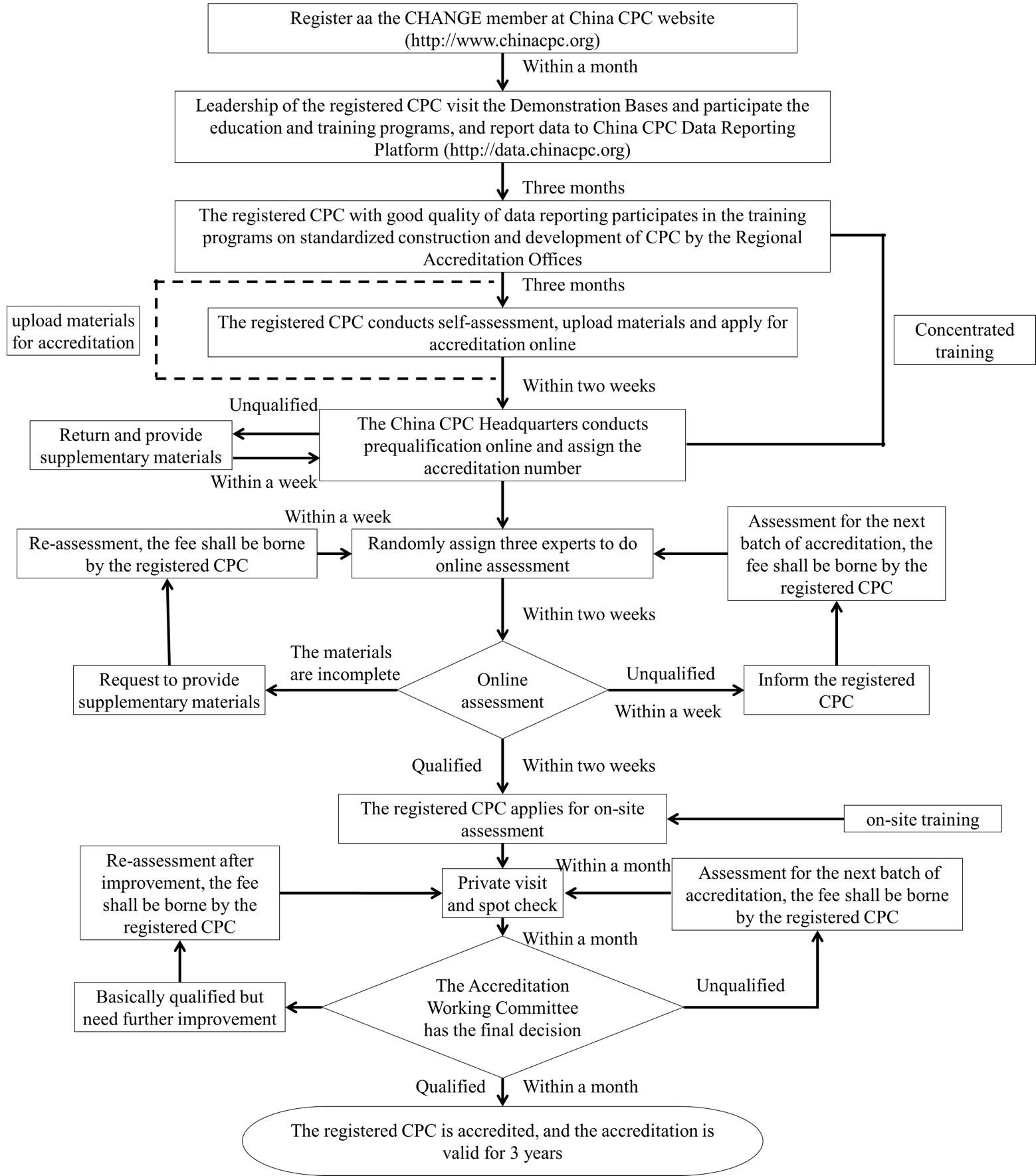


**Appendix Figure 2.** The Chest Pain Center Accreditation Workflow in the CHANGE program

**Appendix Table 1.** The CHANGE registry data elements

| **Category** | **Version 2.0 Data Elements** |
| --- | --- |
| Patient demographics | Age, sex, race, marriage, education, employment, insurance status |
| Medical history and risk factors | Height, weight, hypertension, hyperglycemia, hyperlipidemia, current smoker, family history of heart disease, prior cardiac history, prior revascularization |
| Prehospital treatment | Location of onset, onset date/time, transfer status, arrival date/time, date/time of first medical contact, date/time of first ECG, bypass ED/CCU |
| Presenting features and evaluation | ECG findings, consciousness, heart rate, systolic/diastolic blood pressure, cardiogenic shock, heart failure, Killip class, troponin concentration, serum creatinine, mini-GRACE risk score, preliminary diagnosis |
| In-hospital medication | Antiplatelet agents (aspirin, Clopidogrel/tegrillo), anticoagulant agents (warfarin, unfractionated heparin, low molecular weight heparin, bivalirudin, fondaparinux), intensive statin, β-blockers |
| In-hospital reperfusion strategy | LVEF assessment, Time to sign informed consent, primary PCI date/time (Cath lab activation date/time, Cath lab arrival date/time, door-to-balloon time), thrombolytic date/time, rescue PCI date/time, CABG date/time, recanalization date/time, TIMI class |
| In-hospital outcomes | Heart failure, length of stay, total cost, clinic outcomes (discharge/death/transfer to other hospitals) |
| Hospital Discharge | Principal discharge diagnosis, discharge medicines (DAPT, ACEI/ARB, statin, β-blockers), discharge counseling (Smoking cessation counseling, weight control counseling, blood pressure lowering medications, lipid lowering medications, glucose lowering medications, antithrombotic, follow up scheduling) |

Abbreviation: ECG, Electrocardiograph; ED, emergency department; CCU, coronary care unit; PCI, percutaneous coronary intervention; LVEF, left ventricular ejection fraction; CABG, coronary artery bypass graft; DAPT, dual antiplatelet therapy; ACEI, angiotensin-converting enzyme inhibitor; ARB, angiotensin receptor blocker.

**Appendix Table 2.** Effect of the optimized QI intervention on quality metrics

| Outcomes | ICC | Cases, No (%) | | Cluster-Adjusted | | Primary Analysis | | |  |
| --- | --- | --- | --- | --- | --- | --- | --- | --- | --- |
|  |  | Intervention | Control | Difference  (95% CI) | Odds ratio  or β Coefficient  (95% CI) | | Difference  (95% CI) | Odds ratio  or β Coefficient  (95% CI) | |
| **Service outcome** |  |  |  |  |  | |  |  | |
| Number of admissions |  |  |  |  |  | |  |  | |
| PCI rate |  |  |  |  |  | |  |  | |
| Percentage of EMS transfer |  |  |  |  |  | |  |  | |
| Onset-to-FMC time to mean (SD) |  |  |  |  |  | |  |  | |
| Door-to-balloon time to mean (SD) |  |  |  |  |  | |  |  | |
| FMC-to-device time to mean (SD) |  |  |  |  |  | |  |  | |
| Percentage of onset-to-FMC time ≤60 min |  |  |  |  |  | |  |  | |
| Percentage of Call-to-EMS time ≤15 min |  |  |  |  |  | |  |  | |
| Percentage of Door-to-balloon time ≤60 min |  |  |  |  |  | |  |  | |
| Percentage of FMC-to-device time ≤90 min |  |  |  |  |  | |  |  | |
| **Patient outcome** |  |  |  |  |  | |  |  | |
| In-hospital mortality |  |  |  |  |  | |  |  | |
| 1-year mortality |  |  |  |  |  | |  |  | |
| 1-year complication rate |  |  |  |  |  | |  |  | |

Abbreviations: QI: quality improvement; PCI: percutaneous coronary intervention; EMS: emergency medical service; FMC: first medical contact;

**Appendix Table 3.** Comparison of reach and adoption of the optimized QI initiatives between pre- and post-intervention

| Outcomes | Control period | | Intervention period | | Adjusted Difference  (95% CI) | Adjusted Odds ratio  (95% CI) | P |
| --- | --- | --- | --- | --- | --- | --- | --- |
|  | Number of cases | Proportions  (95% CI) | Number of cases | Proportions  (95% CI) |  |  |  |
| **Implementation outcome -Reach** |  |  |  |  |  |  |  |
| Number of STEMI patients visits |  |  |  |  |  |  |  |
| Number of community residents receiving health education |  |  |  |  |  |  |  |
| Number of health providers receiving QI initiatives training |  |  |  |  |  |  |  |
| **Implementation outcome -Adoption** |  |  |  |  |  |  |  |
| Number of community residents attending the initiatives |  |  |  |  |  |  |  |
| Number of health providers attending the initiatives |  |  |  |  |  |  |  |
| Behavior change of healthcare providers - Change score |  |  |  |  |  |  |  |
| Health literacy change of residents - Change score |  |  |  |  |  |  |  |
| Attitude of health facility directors - Degree of acceptance of the initiatives |  |  |  |  |  |  |  |

Abbreviations: QI: quality improvement; STEMI: ST-segment elevation myocardial infarction; CI: confidence interval.

**Appendix Table 4. Tasks, approaches, and activities of monitoring of the project**

| **Tasks** | **Approaches** | **Activities of monitoring** |
| --- | --- | --- |
| Pilot study | Develop CFIR framework | Check records of framework development |
|  | In-depth interviews with key informants | Check transcription, preprocessing, and data coding |
|  | Report on pilot | Check data analysis, and reporting of actionable findings |
| Design of the optimized QI initiatives | Case study | On-site visits |
|  | Qualitative Interviews and formative research | Check interview outlines and train interviewers |
|  | Data analysis and report | Check intervention design and implementation guide |
| Implementation and evaluation of the optimized QI initiatives | Stepped-wedge cluster randomized control trial | Check sampling, randomization, and management protocols |
|  | Hospital-based assessments | On-site visits every 3 months, and check monthly records of data |
|  | Questionnaire survey on healthcare providers | Train investigators, and check questionnaire and data collection |
|  | Community-based household survey | Train investigators, and check questionnaire and data collection |
|  | Key informant interviews | Check interview outlines and train interviewers |
|  | Data analysis and report | Check data analysis, and reporting of actionable findings |
| Development of scale-up of the optimized QI initiatives | Stakeholder-based participatory research | Check representative of participants, data transcription and preprocessing |
|  | Policy dialogue and round-table discussion | Check pre-activity materials, planning, and organizing |
|  | Data analysis and final Report | Check documentation of the results, and reporting of actionable findings |

Abbreviation: CFIR: Consolidated Framework for Implementation Research; QI: quality improvement.

**Appendix Table 5.** Approaches, risk description, and quality control procedures of the project

| **Approaches** | **Risk description** | **Quality control procedures** |
| --- | --- | --- |
| Develop CFIR framework | Poor interpretability of the framework | Strong scientific justification and guidance of best practices in the world. |
| Case study | Incomparability of different cases | Findings of case studies will be synthesized based on the same transferability framework, to ensure the cohesiveness and accuracy. |
| Stepped-wedge cluster randomized control trial | Administrative disapproval | Inclusion of administrator in project process, especially in study design and implementation. Local project office will be set up to manage the process of implementation. |
| Hospital-based assessments | Infeasible or un-acceptable intervention design | Inclusion of all registered hospitals to process from the beginning of related work package. The research team member will monthly monitor the process of implementation. |
| Questionnaire survey on healthcare providers | Poor fidelity in survey | All investigators will be trained before survey according to standard procedures. Double check the data collection and processing. |
| Community-based household survey | Low participation in survey | Sending invitations timely and assuring an official permission for participation. Double check the data collection and processing. |
| Key informant interviews | Incomplete information collection | Well-design of the interview outlines, training of interviewers on communication skills and interview techniques |
| Stakeholder-based participatory research | Poor representative of participants | Collecting contact information of possible stakeholders from the very beginning of the project. Building close and effective communication channels with all parties. |
| Policy dialogue and round-table discussion | Limited reach in dissemination | Provision of clear and attractive information material to the participants. Assuring an official permission for attending dissemination event. |
| Data analysis and report | Bias in interpretation of findings | All measurement tools will be standardized. Experts in public health, clinical medicine, implementation science, statistics, and sociology will be responsible for the quality of data analysis and interpretation. |

Abbreviation: CFIR: Consolidated Framework for Implementation Research.
